# Metastatic renal cell carcinoma to pancreas and gastrointestinal tract: a clinicopathological study of 3 cases and review of literature

**DOI:** 10.1186/s12894-021-00854-z

**Published:** 2021-05-25

**Authors:** Jamshid Abdul-Ghafar, Nasir Ud Din, Ramin Saadaat, Zubair Ahmad

**Affiliations:** 1 Department of Pathology and Clinical Laboratory, French Medical Institute for Mothers and Children (FMIC), Kabul, Afghanistan; 2 Department of Pathology and Laboratory Medicine, Aga Khan University Hospital, Karachi, Pakistan

**Keywords:** Renal cell carcinoma, Clear cell, Rhabdoid, Metastasis, Pancreas, Colon

## Abstract

**Background:**

Renal Cell Carcinoma (RCC) metastasizes in approximately 20–30% cases. The most common sites for metastases are the lungs, bones, liver, and brain. Metastases of RCC in the gastrointestinal tract (GIT) are very rare. Metastatic RCC has a poor prognosis. We herein present a case series of three patients with metastatic disease in the colon, duodenum, and pancreas following complete resection of RCC.

**Methods:**

Hematoxylin and Eosin and immunohistochemical slides of 3 cases of RCC metastatic to GIT were reviewed. These cases were diagnosed between 2002 and 2019 at French Medical Institute for Mothers and Children (FMIC), Kabul, Afghanistan, and Aga Khan University Hospital (AKUH), Karachi, Pakistan. We also present a detailed review of published literature.

**Results:**

We reviewed cases of three patients, two females and one male, with a mean age of 57.3 years (range 40–67 years) who underwent nephrectomy for RCC. They developed metastases in the colon, pancreas, and duodenum, respectively 12–168 months (median time 156 months) following primary tumor resection. The patient with metastatic RCC in colon presented with abdominal pain and constipation. An ulcerated mass was found on colonoscopy 30 cm from the anal verge. Diagnosis of RCC with rhabdoid features was confirmed in both primary and metastatic tumors. The second patient developed a metastatic nodule in the head of pancreatic while the third patient developed metastatic nodules in the duodenum and pancreas which were detected by Computed Tomography (CT) scanning. Histopathological examination confirmed the presence of clear cell RCC in the metastatic nodules in both cases.

**Conclusion:**

Metastatic RCC should be considered in the differential diagnosis of mass in the gastrointestinal (including pancreaticobiliary) tract especially in presence of a past history of RCC. These patients should be screened thoroughly by physical examination and appropriate imaging studies.

## Background

Renal cell carcinoma (RCC) accounts 85% of renal neoplasms and 2% of all malignant neoplasm of the body. Commonly RCC develops in the cortical region of the kidney. RCC is more common in males [1]. Among many different histologic variants of RCC, clear cell RCC is the most common histological type, around 75–80% of RCCs cases [2]. Papillary and chromophobe RCC types comprise approximately 10% and 5% of all RCC tumors, respectively. Papillary and chromophobe RCC are less aggressive while collecting duct carcinoma and unclassified RCC are rarer and more aggressive [3]. RCC with rhabdoid features generally are more aggressive with rapid growing and causing high mortality rate. Studies found that median survival rates ranged from 8 to 31 months and metastasis occurs in up to 70% of rhabdoid cases [4–6]. Overall, RCC metastasize in approximately 25% cases. Lungs, bones, liver and brain are the most frequent sites where RCCs are metastasizing. However, gastrointestinal tract (GIT) is not the common site of metastatic RCCs and rarely reported [7]. RCC has the potential to metastases to the distant organ after many years [8]. Metastatic RCC has poor prognosis with a 5-year survival of 0–18% in patients with untreated metastatic disease [9]. Thomason et al. in 1991, reported the first case of RCC metastasizing to colon and based on our extensive literature review, to date 26 cases have been reported in literature [10–35] (Table 1).

**Table 1:** Reported cases of metastatic renal cell carcinoma in colorectum

Number cases [citation]	Age/sex	Symptoms	Site	Interval	Histology
1. Ruiz et al. [10]	73 Y/M	Obstruction	Transverse colon	11 years	Clear cell
2. Thomason et al. [11]	71 Y/M	Bowel changes habit	Descending colon	17 years	Clear cell
3. Zerbib et al. [12]	64 Y/M	Bleeding	Descending colon	At time of diagnosis	Clear cell
4. Tokonabe et al. [13]	83 Y/M	Melena	Transverse colon	7 years	Clear cell
5. Avital et al. [14]	72 Y/F	NM*	Right colon	5 years	Clear cell
6. Misu et al. [15]	67 Y/M	Constipation	Sigmoid colon	4 years	Clear cell
7. Sawh et al. [16]	53 Y/M	Pain and bleeding	Anus	9 years	Clear cell
8. Rosito et al. [17]	55 Y/M	Hematochezia	Distal rectum	9 months	Clear cell
9. Mori et al. [18]	71 Y/M	Fecal occult blood	Ascending colon	At time of diagnosis	Clear cell
10. Dellon et al. [19]	70 Y/M	Hematochezia	Rectum	NM	NM
11. Víctor Edmundo et al. [20]	60 Y/M	Hematochezia	Splenic flexure	8 years	Clear cell
12. Yetkin et al. [21]	60 Y/M	Dyspepsia and pain	Hepatic flexure	5 years	Clear cell
13. Jadav et al. [22]	67 Y/F	Abdominal pain	Transverse colon	9 years	Clear cell
14. Chetty [23]	92 Y/M	Asymptomatic	Ascending and sigmoid colon	17 years	Clear cell
15. Zhao et al. [24]	54 Y/M	Hematochezia	Ascending colon	At time of diagnosis	Chromophobe
16. Milović et al. [25]	63 Y/M	Bloating	Sigmoid colon	10 Months	NM
17. Tsamis et al. [26]	64 Y/M	Anemia	Ascending colon	5 Months	NM
18. Davies et al. [27]	76 Y/M	NM	Perianal	7 years	Clear cell
19. Elaine Vo et al. [28]	67 Y/M	Hematochezia	Rectosigmoid	9 years	Clear cell
20. Yuji Maehata et al. 2016 [29]	61 Y/M	hematochezia	Rectum	NM	Clear cell
21. Berry et al. [30]	75 Y/M	Abdominal pain	Sigmoid colon	At time of diagnosis	Papillary
22. Guoyang Zheng et al. [31]	65 Y/M	NM	Rectum	10 years	Clear cell
23. Simon Ouellet et al. [32]	78 Y/M	Painless bleeding	Rectum	NM	Clear cell
24. Valere et al. [33]	33 Y/F	Abdominal pain	Descending colon	3 years	Sarcomatoid
25. Subaşı et al. [34]	63 Y/M	hematochezia	Splenic flexure	5 years	Clear cell
26. Kataoka et al. [35]	65 Y/M	Hip joint pain	Ascending colon	1 year	Clear cell
27. Current case	46 Y/F	Pain and constipation	Descending colon	1 year	Rhabdoid RCC

^*^NM not mentioned

We present three cases with colonic, duodenal and pancreatic metastases following complete resection of RCC of clear cell and rhabdoid types.

## Methods

The Surgical Pathology files of Section of Histopathology, Departments of Pathology and Clinical Laboratory, French Medical Institute for Mothers and Children (FMIC) and Aga Khan University Hospital (AKUH) were searched for cases of metastatic RCC reported between 2002 and 2019. The histological slides were reviewed by two of the principal authors (JAG and NU). All Hematoxylin and Eosin (H&E) and immunohistochemical (IHC) stained slides of the primary and metastatic tumors were reviewed. Since this was a retrospective study involving review of H&E slides, chart review and analysis of radiological findings, ethical approval was not sought. Informed consent was obtained from patients in whom follow up was available.

## Result

A total 3 cases were reported, one case at FMIC and two cases at AKUH.

**Fig. 1:**
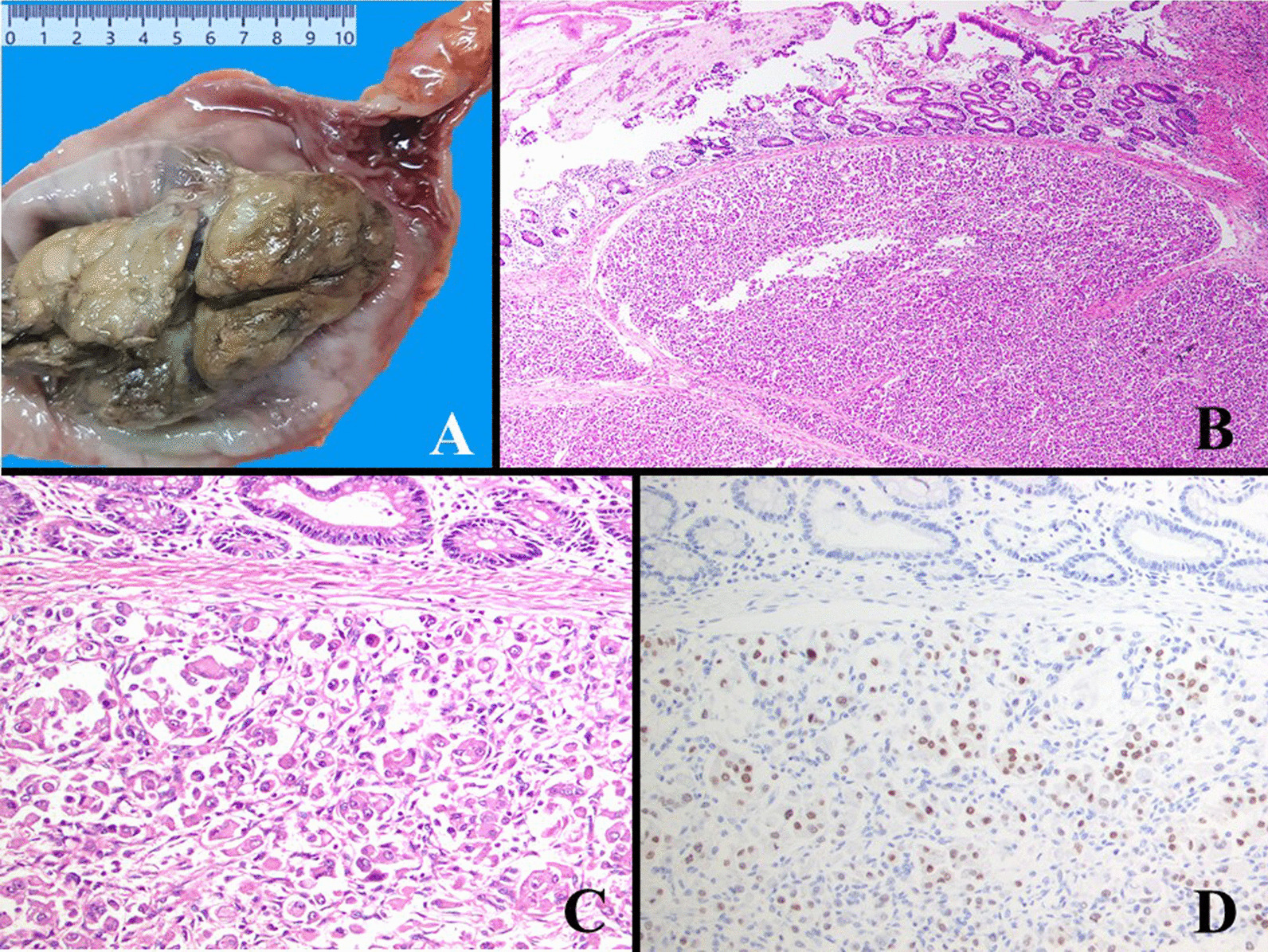
Grossly after opening of the segment of rectum, there was an exophytic mass with lobulated surface (**a**). On microscopic low power, a submucosal neoplasm arranged in sheets, alveolar and rhabdoid patterns was seen (**b**). The neoplastic cells were pleomorphic having abundant eosinophilic cytoplasm, enlarged hyperchromatic eccentric nuclei and prominent nucleoli (**c**). IHC stains were positive for PAX8 (**d**)

*Case 1* A 40-year-old woman with a history of persistent abdominal pain and constipation along with bleeding per rectum was referred by her physician to a gastroenterologist at a private hospital. She had these symptoms for three months. A colonoscopy examination was performed and revealed an ulcerated growth at 30 cm from the anal verge. A biopsy from the ulcerated mass was performed during the colonoscopy procedure and reported as hematoma. No dysplasia or malignancy were observed. Patient gave history of radical nephrectomy one year ago performed at another private hospital. The nephrectomy specimen was diagnosed on histopathological examination as urothelial carcinoma in a private pathology laboratory. Gastroenterologist was not satisfied with diagnosis and referred the patient to a surgeon at his hospital. The patient underwent segmental resection of the colon and specimen was sent to us for histopathological examination. The specimen consisted of a 54 cm segment of colon. On opening, an exophytic mass with nodular surface measuring 11 × 6 × 4 cm was seen in the distal portion of the segment (Fig. 1a). Representative sections from the mass were submitted for histopathological examination. On histopathological examination, sheets of large pleomorphic neoplastic cells with large amount eosinophilic cytoplasm, enlarged, pleomorphic, hyperchromatic eccentric nuclei and visible nucleoli (rhabdoid pattern) were seen (Fig. 1b, c). In areas, tumors cells showed an alveolar arrangement. The classic histologic pattern of RCC was not observed. IHC stains were performed on neoplastic cells and showed positivity for cytokeratin (CK) AE1/AE3 and Cam 5.2, SATB2, vimentin and PAX8 (Fig. 1d). The tumor cells did not show reactivity with TFE3, desmin, MyoD1, S100 protein, anti-smooth muscle actin (ASMA), HMB-45, Melan A, CK7, CK20, CDX2, CD34, CD117, synaptophysin, and Hepar-1. Based on above IHC staining profile a final diagnosis of RCC with rhabdoid features metastasizing to colon was rendered. Tissue slides of the previous nephrectomy specimen were also re-evaluated, and the microscopic findings concur with diagnosis of RCC with rhabdoid features. The pathological stage of radical nephrectomy was pT2aNxMx with tumor size of 7.5 cm in the greatest dimension. Patient died of disease 4 months after the diagnosis of colonic metastasis of RCC.

**Fig. 2:**
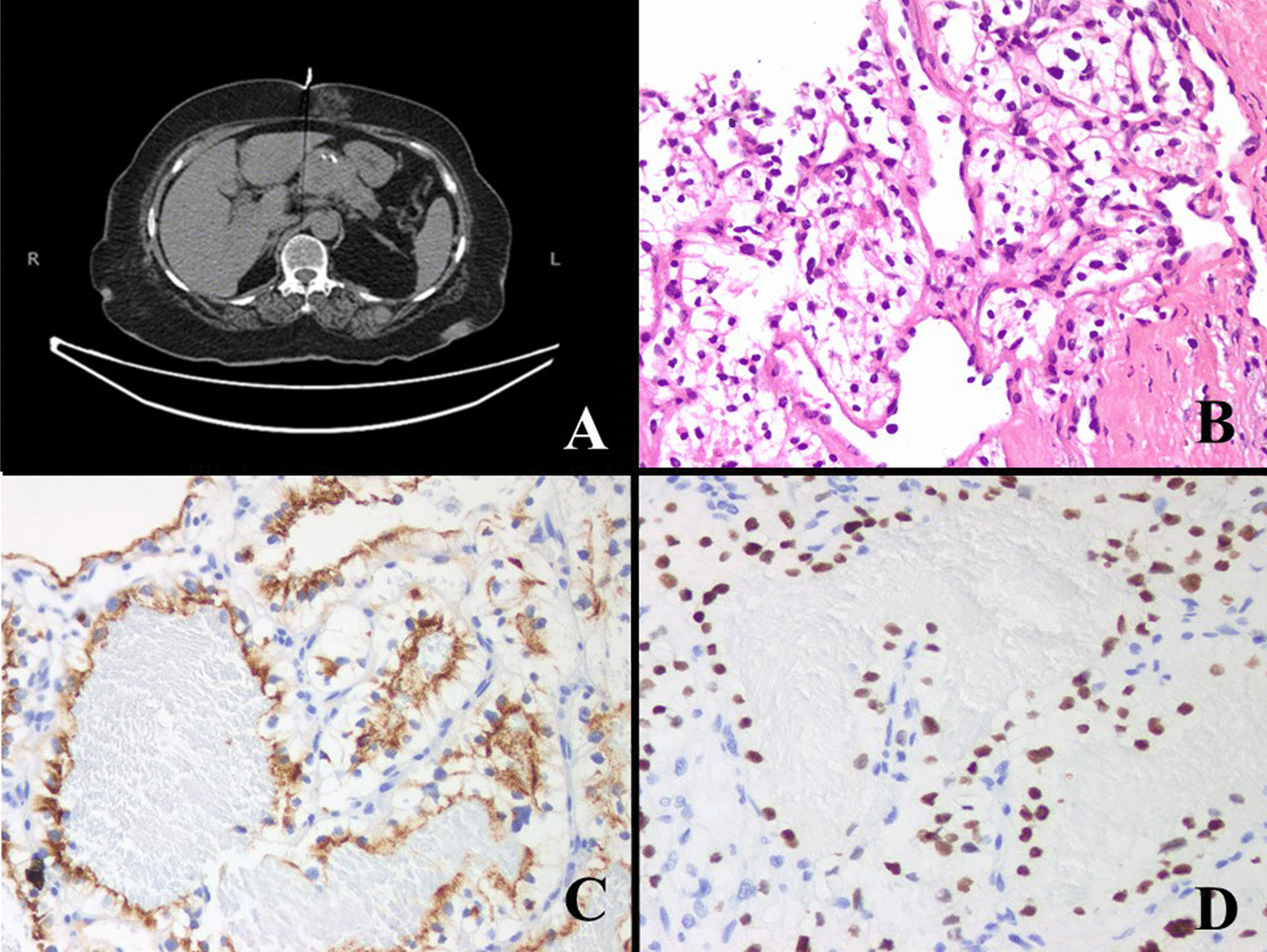
CT scan findings reveals a mass in pancreatic head (**a**). Microscopically, the tumor cells were large and have distinct cell borders with central round hyperchromatic nuclei and abundant clear cytoplasm (**b**). IHC stains were positive for RCC (**c**) and PAX8 (**d**)

*Case 2* A 67-year-old woman visited our hospital with complain of persistent abdominal pain. Her Computed Tomography (CT) scan findings showed a mass in pancreatic head (Fig. 2a). Suspicion of a neuroendocrine tumor was raised. The patient had a history of right radical nephrectomy performed 14 years ago which was diagnosed as clear cell RCC, Fuhrman’s nuclear grade II and staged pT1bNxMx. The tumor measured 5.5 × 5 × 4 cm. CT guided core needle biopsy of the pancreatic head mass was performed and sent to us for histopathological examination. Grossly three linear cores were found. Microscopically, a neoplastic lesion was seen. Tumor cells were large with distinct cell borders, central round hyperchromatic nuclei, visible nucleoli and abundant clear cytoplasm (Fig. 2b). Special stain PAS ± D reveal abundant cytoplasmic glycogen in the tumor cells. On IHC, tumor cells expressed RCC, PAX8, CD10 and Vimentin (Fig. 2c, d) and were negative for CK7 and CK20. Diagnosis of metastatic clear cell RCC was confirmed. Patient refused further treatment. Seven months later, she developed shortness of breath and her condition deteriorated. On admission, her chest x-ray showed pleural effusion and infiltrates in both lungs suggestive of metastatic disease. She died after a week.

*Cases 3* A 65-year-old man with a history of right radical nephrectomy 11 years ago for a renal mass, was on regular follow up with CT scan abdomen. The renal mass was measured as 9.5 × 5 × 4cm and was diagnosed as clear cell RCC, Fuhrman’s nuclear grade II and staged pT2aNxMx. Recently, the follow up CT scan revealed single nodules in duodenum and pancreas. Metastatic disease was suspected, and Whipple’s resection was done, which consisted of part of stomach, duodenum, pancreas and gallbladder. Grossly, a mass measuring 4 × 3.5 × 3.5 cm was found in duodenum while another mass measuring 3 × 2.5 cm was identified in the pancreas. Representative sections from both nodules were submitted for histopathological examination. Microscopic examination of both nodules revealed a neoplastic lesion arranged in nests and cords separated by thin vascular septae. The neoplastic cells were polygonal in shape with clear cytoplasm and rounded, hyperchromatic nuclei. The cells were diffusely positive for cytoplasmic glycogen highlighted on special stain PAS ± D. A panel of IHC stains was performed. The neoplastic cells were positive for CK AE1/AE3, CK Cam 5.2 and Vimentin and negative for Chromogranin A, Synaptophysin and S100. In view of clinical history, morphology and IHC profile, diagnosis of metastatic clear cell RCC was made. Patient died a week after surgery due to postoperative complications.

## Discussion

RCC is the most common type of renal neoplasms in patients above 50 years of age. It is more common in males with a male to female ratio of 2:1 [1]. In about 20–30% cases, primary RCC and its metastasis to the distant organ will be diagnosed in the same time, which is called synchronous presentation. 20% of RCC patients with non-metastatic disease at diagnosis will later develop metastases during follow-up (metachronous presentation) [36].

In very rare cases of RCC, metastatic involvement of GI tract can be observed. Metastasize to any part of GI tract can occur but metastasis to colonic is much rarer compared to gastric and to small intestine [37]. Colon usually involved by metastatic cancers from breast, stomach and malignant melanoma of the skin [38]. RCC not infrequently metastasizes to pancreas, RCC metastases to pancreas account for 2–5% of all pancreatic neoplasms and metastatic RCC is the most common secondary neoplasms in the pancreas followed by malignant melanoma and colorectal carcinoma [39]. Metastasis of RCC to periampullary region is very rare and can occur several years (6.5–12) after resection of primary renal tumor. Metastasis 32 years following previous resection has been reported [40]. In these patients overall, 5-year survival rates are less than 10% but surgical resection can improve 5-year survival rates up to 88%. Improvement in survival is even seen in multi-focal metastasis [40]. A 5-year survival rate of 75% was reported in a retrospective study of surgically treated pancreatic metastases in ten patients, with the longest survivor at 117 months after resection. Therefore, the study suggests that RCC metastasis to pancreas should be managed aggressively with complete resection [41]. European Association of Urology (EAU) suggests that metastasectomy remained, by default, an appropriate local treatment for most sites with the exception of brain and possibly bone metastases, which are frequently treated by stereotactic radiotherapy [42].

In our study, the time for metastases to pancreas following nephrectomy was 14 years in case 2 and 11 years in case 3.

A study reported two cases of metastatic RCC in the pancreas with mean time of metastasis of 41.5 months (0–108). Both patients were males aged 65 and 87 years [43]. RCC metastasis to colon can occur several years following curative resection of the primary tumor or it can occur soon after resection [44]. On reviewing the reported cases of colon-rectal metastasis of RCC (Table 1), one found that most colon metastases occurred after 5 years (maximum 17 years). Only three cases metastasized to colon within one year after resection of primary RCC. Interestingly, all reported cases of metastatic RCC in colon occurred in males and all patients were over 50 years old (53–92 years), while in our case metastatic RCC to colon occurred in a female patient who was below 50 years of age (46 years). In addition, majority of reported metastatic RCC cases in colon presented with rectal bleeding. Therefore, metastatic RCC should be kept in mind in patients with known history of RCC who presented with rectal bleeding. In our case, patients presented with abdominal pain and constipation, probably be due to large size of the mass (11× 66 × 64 cm).

In our study, metastasis to pancreas and pancreatico-duodenal region occurred after 11 and 14 years after resection of primary RCC.

In terms of appearance, we found in published literature cases of metastatic RCC in pancreas have been mostly reported in men (75%) with average age of 65.5 years [41]. Metastases can occur through lymphatic or hematogenous routes or by direct invasion. Large tumor size increases the chances of metastatic spread. However, metastases to lymph nodes and distant organs can occur even in the early stages of RCC [45].

On literature review, not surprisingly, the most common metastatic type of RCC to GI tract is clear cell type, the commonest RCC subtype [46]. However, papillary type, chromophobe type and sarcomatoid type RCC metastasizing to colon have been reported [37, 47, 48].

In our case, metastatic RCC in colon turned out to be RCC with rhabdoid features. Among subtypes of RCC the rhabdoid features is very rare account up to 5% of all RCCs. RCC having rhabdoid features behave more aggressively and usually develops extra renal invasion and has higher potential for distant organ metastases comparing to other subtypes of RCC. Therefore, the prognosis of patients diagnosed RCC with rhabdoid features is very poor [49].

Histologically, rhabdoid cells are characterized by large polygonal cells with large amount pink-eosinophilic cytoplasm and having pleomorphic eccentric nuclei with visible large nucleoli. On IHC staining, rhabdoid cells show positive reactivity with vimentin, epithelial membrane antigen (EMA), cytokeratins and PAX8 but not reactive for desmin, myoglobin, myogenin and Myo D1 [4, 50]. To the best of our knowledge no case of metastatic RCC with rhabdoid features has been reported in literature and our case represents the first case of RCC with rhabdoid features metastasizing to colon. RCC with rhabdoid features should be differentiated from other malignant tumors having rhabdoid cell morphology. Alveolar Soft Part Sarcoma (ASPS) has the similar histologic morphology, the cells are arranged in alveolar pattern and are polygonal with eosinophilic granular cytoplasm. ASPS can rarely occur or metastasize to colon. On IHC staining, Myo D1, TFE3 and vimentin widely expressed among ASPS cells [51], while these stains were not expressed in our case. Another malignant neoplasm which has the similar morphology is pleomorphic rhabdomyosarcoma. Pleomorphic rhabdomyosarcoma having large pleomorphic and polygonal cells of rhabdomyoblasts. On IHC staining, the neoplastic cells in pleomorphic rhabdomyosarcoma show positive reactivity with myogenin, desmin, smooth muscle actin (SMA) and vimentin. The cells of RCC with rhabdoid features do not express these markers [52].

It is quite difficult to distinguish metastatic RCC having rhabdoid features from ASPS and pleomorphic rhabdomyosarcoma only on histologic morphology. In our case, IHC profile helped to diagnose the RCC with rhabdoid features over ASPS and pleomorphic rhabdomyosarcoma. Our case was negative for TFE3, Myo D1, myogenin, desmin and positive for renal cell markers such as PAX8 and Cam5.2. In addition, history of nephrectomy and review of previous nephrectomy slides further supported the diagnosis of metastatic RCC with rhabdoid features.

Metastasis to colon in the current study occurred in less than one year following nephrectomy. This interval is much shorter than in the literature. This is probably because RCC with rhabdoid features is an aggressive type of RCC with high potential for rapid early metastasis.

Few differential diagnoses should also be kept in mind RCC metastasizing to pancreas. These include solid variant of serous cystadenoma of pancreas whose cells may have clear cytoplasm and well-defined cell borders. However, hemorrhage and nuclear atypia are rare. In contrast, metastatic RCC usually demonstrates foci of hemorrhage and nuclear atypia. Expression of PAX8 and CD10 by RCC cells is important for confirmation [53, 54]. It should also be kept in mind that cases of metastatic RCC to pancreas with underlying primary pancreatic microcystic serous cystadenoma have been reported and may cause difficulty in identifying metastatic RCC [55]. In such cases, careful microscopic evaluation along with IHC staining can differentiate the two tumors as serous cystadenoma cells do not express CD10 and PAX-8 and RCC but are positive for CK7 while RCC cells are negative for CK7 and positive for CD10 and PAX-8. Solid pseudo-papillary neoplasm (SPN) of the pancreas with clear cell features is another entity that must be differentiate from metastatic RCC. However, this tumor occurs mostly in young age and tumor cells are positive for synaptophysin and B-catenin [56].

PEComa should be also considered in differential diagnosis, although, it rarely occurs in pancreas and colon but, the similar histologic morphology can be challengeable. PEComa has polygonal to round cells and are having clear cytoplasm. To surely differentiate it from RCC with rhabdoid features, IHC staining is required. Tumor cells in PEComa strongly express ASMA and HMB45 stains while these IHC stains are negative for RCC cells [57]. Radiology is of little significance in diagnosing skin metastasis. However, metastasis of RCC to other locations can be recognized [58].

Histology is the gold standard for diagnosing metastasis and should be performed in all situations with adequate precautions. Light microscopy alone cannot help much in differentiating metastatic RCC from other clear cell tumors and IHC is required for accurate diagnosis [59].

## Conclusion

Metastatic RCC especially of clear cell subtype to the colon and pancreas should be considered in the differential diagnosis especially if there is a previous history of RCC or nephrectomy. Patients with lower GI symptoms or mass, particularly those patients with the past history of renal mass or nephrectomy should be screened thoroughly by physical examination and imaging modalities for metastases to gastrointestinal tract (including pancreaticobiliary tract) and other locations.

## Data Availability

Data and materials of this work are available from the corresponding author on reasonable request.

## Abbreviations

RCC: renal cell carcinoma
CT scan: computed tomography scan
GIT: Gastrointestinal track
FMIC: french medical institute for mothers and children
AKUH: Aga Khan University Hospital
H&E: Hematoxylin and Eosin
IHC: immunohistochemical
CK: Cytokeratin
ASMA: anti smooth muscle actin
EMA: epithelial membrane antigen
ASPS: Alveolar Soft Part Sarcoma
